# Corrections

**Published:** 1870-01

**Authors:** 


### CORRECTIONS.

There were a few misprints in the article "Miscellanies," Dec' No—page 417, 14th line from top, "with the understanding," should read without the understanding. Page 419, first line, new paragraph, for "But these are pictures," it should be, there are pictures.

In the December No. of the Register, page 516, the article credited to Dr. Parker, should have been credited to Dr. Wm. P. Barker, Grand Rapids, Mich.

